# Obituary

**Published:** 1884-04

**Authors:** 


### OBITUARY.

Professor Geovanni Battista Ercolani, formerly Director of the Veterinary College of Turin, died at Bologna on the 16th of last November at the age of 64. He was a great patriot as well as scientist, and the profession throughout the world bewails his loss.

Professors Delwart and Thiernesse, formerly directors of the Brussels Veterinary School, both died recently.

Valentine Delwart was born in 1801 and studied veterinary medicine at Alfort, in 1833 he was called to be a Professor at the Belgian School of which he was director from 1860 to '67.

Theodore August Thiernesse was born in 1812, and graduated from the Brussels school. In 1847 he was made a professor, and 1867, director of the same, which position he held until recently. These two Veterinarians were the founders and diligent workers upon the "Annals of Veterinary Medicine," which has been in existence for 32 years; their death has been no inconsiderable loss to veterinary medicine in Belgium.

